# ﻿Redescription of Tachyura(s. str.)ferrugata (Reitter, 1895) (Coleoptera, Carabidae), with the comments on the availability of varieties described by Johan Reinhold Sahlberg in Tachyina

**DOI:** 10.3897/zookeys.1233.145545

**Published:** 2025-03-31

**Authors:** Tomáš Kopecký, Jan Bezděk, Jaakko Mattila

**Affiliations:** 1 Department of Zoology, Fisheries, Hydrobiology and Apiculture, Mendel University in Brno, Zemědělská 1, 613 00 Brno, Czech Republic Mendel University in Brno Brno Czech Republic; 2 Entomology Team, Zoological Museum, Finnish Museum of Natural History, Pohjoinen Rautatiekatu 13, 00014 Helsinki, Finland Finnish Museum of Natural History Helsinki Finland

**Keywords:** Bembidiini, museum collections, Palaearctic Region, Tachyina, taxonomy

## Abstract

Tachyura(s. str.)ferrugata (Reitter, 1895) is redescribed and figured in detail, including male and female genitalia, and a lectotype is designated. Photographs of the type specimens, including labels, are presented. The female gonocoxites are figured for the first time. A review of varieties described by Johan Reinhold Sahlberg in Tachyina is presented, with comments on their availability. The following varieties are treated as unavailable infrasubspecific names: Tachys (Tachyura) quadrisignatus var. caramanicus J.R. Sahlberg, 1913, Tachys (Tachyura) sexstriatus var. brunneicollis J.R. Sahlberg, 1913, and Tachysscutellarisvar.obscurus J.R. Sahlberg, 1913.

## ﻿Introduction

Reitter (1895) described *Tachysferrugatus* Reitter, 1895 from Akbes (= Akbez municipality), which was at that time a Syrian territory and is now part of Hassa District, Hatay Province, Turkey. In the short original description, the number of type specimens is not mentioned, although there is a note that the species was common at the locality. However, only one syntype, designated here as the lectotype, was found in Reitter’s collection deposited in the Hungarian Natural History Museum, Budapest (Kopecký 2009).

Winkler (1924) classified *Tachysferrugatus* in the subgenus Tachyura Motschulsky, 1862. However, Csiki (1928) and Lorenz (1998) did not assign it to a subgenus. In the Catalogue of Palaearctic Coleoptera, Kopecký (2003) included this species in the nominal subgenus of *Tachyura* based on examination of the type specimen (although this was not stated) and based on following characters: body arched, the elytra sloping towards the apex from the posterior third at an angle of 40–50° (in lateral view), the shape of male genitalia resembling “inverted knife blade” with a sclerotized lower part and a distinctly notched hook-like sclerotized tip, the first three humeral punctures are close to each other, the fourth puncture is clearly distant from them, the average ratio of the elytral length and width between 1.4–1.7. Finally, Kopecký (2009) published new distributional data and synonymized *Tachysschuberti* Jedlička, 1968 with *T.ferrugata*.

The original goals of this work were to present a detailed redescription of *T.ferrugata* and to synonymize Tachys (Tachyura) quadrisignatus var. caramanicus J.R. Sahlberg, 1913 with *T.ferrugata*. However, during the finalization of the manuscript, we discovered that var. caramanicus is an unavailable infrasubspecific name, which led us to check the availability of all the varieties described by J.R. Sahlberg in Tachyina. For the first time, *T.ferrugata* is redescribed and depicted using a vector graphic editor, including line drawings of the aedeagus and female gonocoxites.

## ﻿Material and methods

This article is based on the study of extensive material deposited in the following collections: Hungarian Natural History Museum, Budapest, Hungary (**HNHM**); collection of Kamil Orszulik, Frýdek-Místek, Czech Republic (**KOCF**); collection of Michal Grycz, České Budějovice, Czech Republic (**MGCC**); Finnish Museum of Natural History, Helsinki, Finland (**MZH**); Natural History Museum, Milan, Italy (**NHMM**); National Museum, Prague, Czech Republic (**NMPC**); Naturhistorisches Museum, Wien, Austria (**NMW**); collection of Pavel Vonička, Liberec, Czech Republic (**PVCL**); collection of Tomáš Kopecký, Jablonné nad Orlicí, Czech Republic (**TKCJ**); Zoological Institute of Russian Academy of Sciences, St. Petersburg, Russia (**ZIN**); Museum für Naturkunde, Berlin, Germany (**ZMHB**).

Male and female genitalia were dissected, soaked in potassium hydroxide (KOH) then stained with chorazol black and glued using hydration resin onto glass mounted on a card under the specimens. Adult specimens were photographed using a FinePix S5600, Xiaomi 22101316G, Novex and Eakins binocular microscopes. Aedeagi and gonocoxites were photographed using a Xiaomi 22101316G camera on a VEVOR XSP-36TV microscope. Based on the photographs, precise anatomical line drawings were created with the Inkscape vector graphics editor. Exact label data are cited for all type specimens; backslash (\) separates data on different labels.

A lectotype was designated for *Tachysferrugatus* in accordance with Articles 74 and 76 of the International Code of Zoological Nomenclature (International Commission of Zoological Nomenclature 1999) to preserve stability of nomenclature and fix unique bearer of the name of that taxon.

## ﻿Comparative material

***Tachyssinaiticus* Schatzmayr, 1936**

Egypt • Holotype; “Sinai Wadi Ysla 27.2.35 W. Wittmer [white label] \ Typus [red label] \ T. sinaiticus Schatzm. [white label] \ HOLOTYPUS [red label]”; NHMM.

***Tachysemeritus* Péringuey, 1898**

Republic Of South Africa • 1 paratype; “Graphicus barus [white label] \ Paratypus [red label] \ emeritus Pér. [white label]”; ZMHB.

***Tachysthoracicus* Kolenati, 1845**

Unknown Country • 1 syntype; “Transcauc. [red label] \ var. thoracica Kolent. [white label] \ Lectotypus Tachyurathoracica Kol. Kryzhanovskij det. [red label] \ ZOOLOGICAL INSTITUTE RAS ST. PETERSBURG [white label]”; ZIN.

## ﻿Results and discussion

### ﻿ Tachyura (s. str.) ferrugata

Taxon classificationAnimaliaColeopteraCarabidae

(Reitter, 1895)

3CDDBEDE-2F38-5478-910D-6489772B63BB

Figs 1 , 2

Tachys ferrugatus Reitter, 1895: 79 (original description).
Tachys (Tachyura) quadrisignatus var. caramanicus J.R. Sahlberg, 1913b: 18 (unavailable infrasubspecific name).
Tachys schuberti Jedlička, 1968: 289 (original description).

#### Material examined.

**Figure 1.**
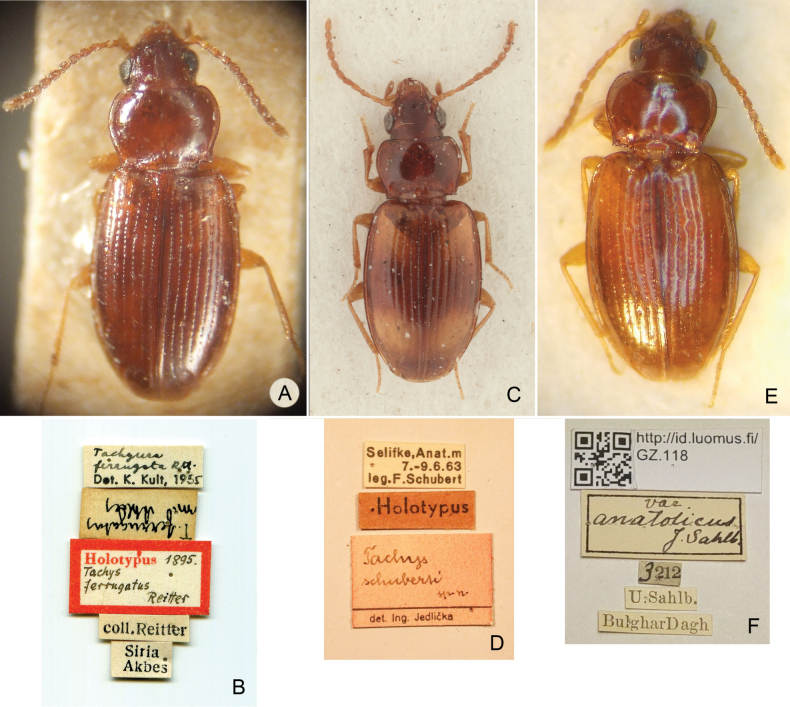
Tachyura(s. str.)ferrugata (Reitter, 1895), type specimens and their labels. **A***Tachysferrugatus* Reitter, 1895, lectotype **B***T.ferrugatus* Reitter, 1895, lectotype, labels **C***T.schuberti* Jedlička, 1968, holotype, female **D***T.schuberti* Jedlička, 1968, holotype, labels **E**T. (Tachyura) quadrisignatus var. caramanicus J.R. Sahlberg, 1913, original specimen **F**T. (Tachyura) quadrisignatus var. caramanicus J.R. Sahlberg, 1913, labels.

***Type material***: Turkey • Lectotype (Fig. 1A, B); “Siria, Akbes [white label] \ coll. Reitter [white label] \ T.ferrugatus m. Akbes [white label] \ Holotypus 1895 Tachysferrugatus Reitter [white label with red frame] \ Tachyuraferrugata Rtt. Det. K. Kult, 1955 [white label]”; HNHM.

***Note on the type material***: Reitter (1895) used the word “häufig” in the description of *Tachyuraferrugata*, meaning that the species was abundant in the type locality, without specifying the number of specimens examined. We found only one syntype in the Reitter collection deposited in the Hungarian Natural History Museum in Budapest (Fig. 1A, B). The syntype has a holotype label added by the curator Zoltán Kaszab in the 1960s without any justification. We cannot rule out that additional syntypes exist in other institutions. This is why we find it appropriate to follow Recommendation 73F of the Code for avoidance of assumption of holotype (ICZN 1999) and design the specimen in question as lectotype.

Jedlička (1968) described *Tachysschuberti* from Turkish localities “Selifke” (holotype) and “Namrum” (3 paratypes in coll. Schubert in NMW). The holotype (Fig. 1C, D) and 1 paratype are deposited in NMPC. The collections of the father Franz Theodor Adolf Schubert and his son Franz Xaver Schubert are stored in the Natural History Museum Vienna (Groll 2017) and two paratypes were traced there.

In the original description of Tachys (Tachyura) quadrisignatus var. caramanicus, Sahlberg (1913b) mentioned “25 specimina pauca invenit filius Unio” [= 25 specimens collected by his son Unio Johanson Sahlberg] collected in the valley of the Bulghar Dagh mountain range. In MZH, one original specimen of var. caramanicus from Bulghar Dagh, with the collector label “U. Sahlb.” is deposited (Fig. 1E, F). The specimen is labelled “var. anatolicus J. Sahlb.”, which can either be a mistake, or the name was changed during publication process, as no taxon with such name was described by J.R. Sahlberg. This specimen is conspecific with *Tachyuraferrugata*.

***Non-type material***: Syria • 2 spec.; 35 km E Latakia, Slanfah; 1200–1400 m a.s.l.; 29 Apr. 2011; K. Orszulik leg.; KOCF • 14 spec.; Homs env., 10 km N of Crac des Chevaliers, Mashta Al Hilu; 24 Apr. 2011, K. Orszulik leg.; KOCF • 2 spec.; same data as for preceding; TKCJ.

Turkey • 1 ♀ (Fig. 1C, D), holotype of *Tachysschuberti* Jedlička, 1968; “Selifke, Anat.m. 7.–9.6.63 leg. F. Schubert [white label] \ Holotypus [red label] \ Tachysschuberti sp. n. det. Ing. Jedlička [red label]”; NMPC • 3 spec., paratypes of *Tachysschuberti* Jedlička, 1968; “Namrun, Anat.m. 10.5.–3.6.63 leg. F. Schubert [white label] \ Paratypus [red label] \ Tachysschuberti sp. n. det. Ing. Jedlička [red label]”; NMW, NMPC • 1 spec. (Fig. 1E, F) [original specimen of Tachys (Tachyura) quadrisignatus var. caramanicus J.R. Sahlberg, 1913]; Bulghar Dagh; U. Sahlberg leg.; MZH • 1 spec.; Hatay Prov., Amanos Mts., Aslanli Beli; 970 m a.s.l.; 1 Jun. 1991; S. Kadlec leg.”; MGCC • 8 spec.; same data as for preceding; TKCJ • 1 spec.; Harbiya; 22 May 1993; J. Krátký leg.; TKCJ • 1 spec.; Samsun Prov., Yunddagi Mts.; Cakiralan env.; 800–1000 m a.s.l.; 16 Jun. 1998, P. Vonička leg.; PVCL.

#### Redescription.

**Figure 2.**
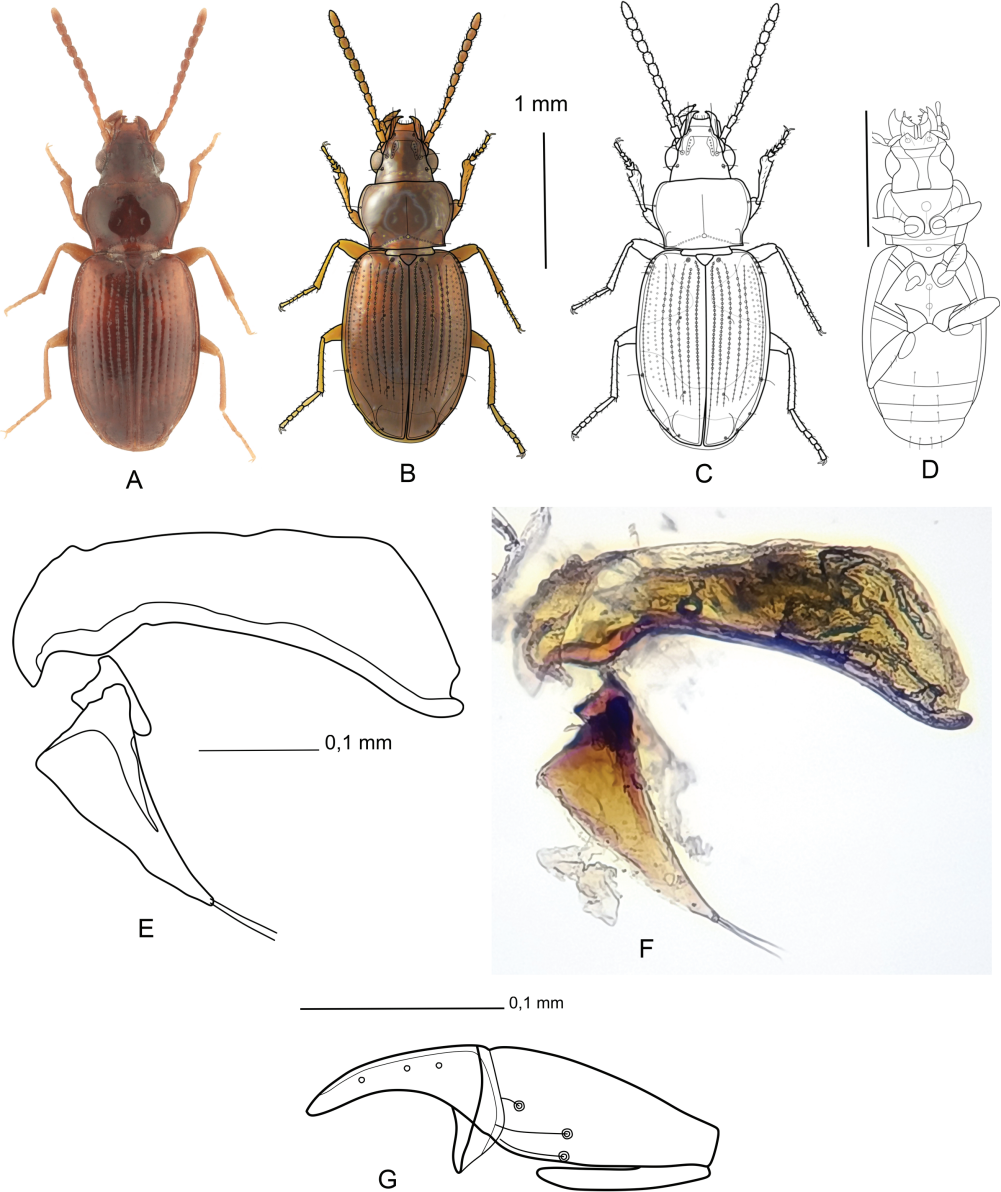
Tachyura(s. str.)ferrugata (Reitter, 1895). **A** dorsal view, male, Turkey: Harbiya **B** dorsal view, male, Turkey: Harbiya, photograph contoured by line drawing **C** dorsal view, male, Turkey: Harbiya, line drawing **D** ventral view, female, Turkey: Bahçe, line drawing **E** aedeagus, Turkey: Harbiya, line drawing **F** aedeagus, Turkey: Harbiya **G** gonocoxite, Turkey: Çevlik.

***Body*** (Fig. 2A–C). In lateral view, elytra more strongly arched, in anterior third slightly lowered, highest in posterior third, from which they bow to apex at an angle of 40°. Body length 2.38–2.50 mm, body width 0.88–0.95 mm.

***Coloration.*** Dorsal side rusty shiny, not iridescent. Each elytron with indistinct traces of apical and humeral pale rust spots. Antennae rusty, antennomeres I, II and half of III pale rust. Legs pale rusty. Ventral part of head, prothorax, mesothorax and metathorax rusty, epipleuron of pronotum and elytra pale rusty, maxillary palpomeres I and II pale rusty, III pale, labial palpomeres I and II pale rusty, III pale.

***Head.*** One-third narrower than pronotum, one quarter wider than long, eyes slightly prominent; labrum with convex anterior margin and with six short setae; clypeus with two punctures on anterior margin each one bearing long seta; frontal furrows double; external frontal furrows start near frontoclypeal suture, then sinuous to anterior setiferous punctures near eyes; internal frontal furrows broad, short and punctate, starting from clypeus and ending internally to anterior setiferous punctures; anterior setiferous punctures located on internal edges at anterior third of eyes, posterior setiferous punctures located on internal edges in posterior quarter of eyes; mandibles sharp, with one little tooth on internal edge; maxillae small, sharp, with two teeth and number of setae; antennomeres I–IV more than twice as long as wide, V–VI more than 1.5 times as long as wide, following antennomeres as long as wide, antennomere I with two setae, antennomeres II with four setae, rest of antennomeres with many setae.

***Pronotum.*** 1.4 times as wide as long, widest before middle, lateral margins widely bent, except basal fifth subparallel; anterior margin nearly straight; posterior margin nearly straight, lateral parts of posterior margin slightly sinuate; anterior angles rounded; posterior angles sharp, each with posterolateral setiferous puncture bearing long seta; lateral margins bordered, each before middle with anterolateral setiferous puncture bearing long seta; basal humps indistinct; basal impressions acutely depressed, leading obliquely from posterior angle towards centre, each impression with ten punctures; area between basal impressions with deep median puncture; median line fine, impressed, beginning at anterior pronotal quarter and ending in median puncture of basal impression; surface without visible microsculpture at 50× magnification.

***Elytra.*** Oval, 1.4 times as long as wide, with broad margin, without humeral teeth, widest at middle; each elytron with eight striae and without scutellar strioles, striae I–V and VIII well visible, with deep punctures, striae VI and VII punctiform (formed only by punctures), stria I begins next to scutellum and passes along suture to apex where it is connected with similarly prolonged stria VIII, which sinuate subapically, striae II–VII begin in anterior tenth and end in apical third of elytra; apical striola long, bent and directed towards stria III; surface without visible microsculpture at 50× magnification; humeral edge of the elytron passes humerus in form of short backward stria; each elytron with following setiferous punctures bearing long setae: large parascutellar puncture, four posthumeral umbilicate setiferous punctures, the first three at distance of their width, the fourth 1.5 times its diameter distant from third; one umbilicate puncture before apical third of elytral margin, two umbilicate puntures on elytral margin before apex; apical pore before half-length of apical striola at level of stria IV, two discal pores on inner edge of stria III, first in anterior third, second in posterior quarter.

***Legs.*** Profemora with four setae on dorsal side, two long setae on anterior margin of ventral side and two long setae in middle of ventral side; outer edges of frontal parts of protibiae obliquely arcuately cut, with three spines, one spine on anterior margin interiorly next to protarsomere I, one spine on anterior margin exteriorly next to protarsomere I and one bigger spine on interior margin of ventral side; protibiae on exterior margin of dorsal side and in ventral side with row of setae; first two protarsomeres in male slightly serrated and extended to sides, each protarsomere on dorsal ventral sides with two setae, protarsomere I 1.5 times as long as wide, protarsomeres II–IV as long as wide, protarsomere V 3.0 times as long as wide, claw falcate. Mesofemora with some setae on anterior margins of dorsal and ventral sides; mesotibiae with rows of setae on dorsal and ventral sides; mesotibiae at apical margin with three spines, two spines interiorly next to mesotarsomere, first in dorsal side and second on ventral side, one spine exteriorly next to mesotarsomere in dorsal side; mesotarsomeres I and V 3.0 times as long as wide, mesotarsomere II 1.5 times as long as wide, mesotarsomere III–IV as long as wide, all mesotarsomeres with two setae on dorsal and ventral sides, claw falcate; metafemora with some setae on anterior margins of dorsal and ventral sides; metatibiae with rows of setae on dorsal and ventral sides; metatibiae at apical margin with three spines, two spines interiorly next to metatarsomere, first in dorsal side and second on ventral side, one spine exteriorly next to metatarsomere in dorsal side; metatarsomere I 4.0 times longer as wide, metatarsomeres II–III 1.5 times as long as wide, metatarsomere IV as long as wide and metatarsomere V 3.0 times as long as wide, all metatarsomeres with two setae on dorsal and ventral sides, claw falcate.

***Ventral surface*** (Fig. 2D). Mentum with deep incision on front edge, anterior margin on each side next to incision pointed, in centre of incision with small tooth, surface below incision on each side with one setiferous puncture; maxillary palpomeres I and II 2.5 times as long as wide, with tiny setae, maxillary palpomere III small and narrow, twice longer as wide and without setae; labial palpomere I narrow, 4.0 times as long as wide, without setae, labial palpomere II 1.5 times as long as wide, kidney shaped, with setae, labial palpomere III small and narrow, 3.0 times as long as wide and without setae; anterior part of gula laterally widened; anterior part of prosternum wide, tapering posteriorly, in middle with pore, prosternal projection rounded axe-shaped; procoxa rounded; procoxal trochanter 1.5 times as long as wide; mesosternum triangular; mesepisternum 1.5 times as long as wide; mesepimeron very narrow, attached laterally to mesepisternum, almost indistinct; mesocoxa rounded; mesotrochanter 2.0 times as long as wide, pointed at end; metepisternum wide, elongate triangular; metepimeron narrow, almost indistinct; metasternum diamond-shaped, in middle with two pores in fine grove; metacoxa horn-shaped; metatrochanter elongate rounded, 2.5 times as long as wide; abdomen with six visible ventrites, ventrite I narrow, widened from centre to sides, ventrites II–III fused, laterally with indistinct sutures, ventrites III–V with two setiferous punctures in middle, ventrite VI with four setiferous punctures in middle.

***Aedeagus*** (Fig. 2E, F). Length 0.396 mm, evenly curved from base to apex in lateral view; superior margin with three shallow depressions and strong incision before apex; apex rounded and strongly sclerotized on underside; inferior margin slightly rounded, sclerotized along its entire length, internal structure not sclerotized; length of right paramere 0.223 mm, more sclerotized at base, with two long setae at apex.

**Female.** Length of gonocoxite 0.204 mm, from base to middle of amphora shape, basal gonocoxite on ventral side with two long apical setae, and one medial seta, apical gonocoxite separates by abdominal collar from basal one, formed on ventral side with tooth-shaped, the anterior part sickle-shaped with three pores (Fig. 2G).

***Bionomy.*** The species lives on sandy gravel banks of streams, from lowlands to mountains.

#### Differential diagnosis.

In the East Mediterranean and the Near East, Tachyura(s. str.)ferrugata can be mixed with T.(s. str.)confusa Coulon & Felix, 2011, T.(s. str.)emerita (Péringuey, 1898), T.(s. str.)sinaitica (Schatzmayr, 1936), and T.(s. str.)thoracica (Kolenati, 1845). Therefore, we list differential characters that help to distinguish the above five species (Table 1).

**Table 1.** Differential characters for species similar to *Tachyuraferrugata*.

Species (*n* = 10)	Body length (mm)	Elytra length / width (mean)	Dorsal striae	Pronotum width / length (mean)	Pronotum shape	Eye length / width (mean)
*T.ferrugata*	2.38–2.50	1.55	Eight strongly punctured dorsal striae, striae I–V and VIII well visible, with deep punctures, striae VI and VII formed only by punctures	1.56	Narrowed to apex, lateral parts of posterior margin slightly sinuate, posterior angles, sharp	1.67
*T.emerita*	1.83–2.08	1.51	Eight punctured dorsal striae, striae I–IV, with punctures, striae V– VIII formed only by punctures	1.94	Slightly narrowed to apex, lateral parts of posterior margin little sinuate, posterior angles obtuse	1.55
*T.thoracica*	2.00–2.45	1.57	Five finely punctured dorsal striae	1.37	Narrowed to apex, posterior margin little sinuate, posterior angles obtuse	1.43
*T.sinaitica*	2.00–2.23	1.65	Five finely punctured dorsal striae	1.33	Narrowed to apex, lateral parts of posterior margin slightly sinuate, posterior angles, sharp	2.00
*T.confusa*	1.88–2.05	1.59	Four dorsal striae, striae III in middle well visible and finely punctate, striae IV formed only by fine punctures	1.40	Narrowed to apex, posterior margin not sinuate, posterior angles obtuse	1.67

#### Distribution.

Syria (Kopecký 2009; present paper), Turkey (Reitter 1895; Sahlberg 1913b; Jedlička 1968; Kopecký 2009; present paper), Yemen (Socotra Island) (Kopecký 2009). The occurrence in Greece, reported in the first edition of Catalogue of Palaearctic Coleoptera (Kopecký 2003), was based on erroneously determined specimens of *Tachyurathoracica* (Kopecký 2009). However, the occurrence in Greece was erroneously repeated in the second edition (Kopecký 2017).

##### ﻿Key to the identification of the East Mediterranean and the Near East species related to *Tachyuraferrugata*

**Table:** 

1(2)	Each elytron with four striae, stria III well visible and finely punctate in middle, IV formed only by fine punctures. Pronotum narrowed to apex, posterior margin not sinuate, posterior angles obtuse. Eyes less prominent, mean ratio of eye length / width 1.67. Dorsal side shiny, dark yellow. Elytra without spots. Antennae and legs dark yellow. Body length 1.88–2.05 mm	***T.confusa* Coulon & Felix, 2011**
2(1)	Each elytron with more than four striae	**3**
3(6)	Each elytron with five striae	**4**
4(5)	Eyes flat, mean ratio of eye length / width 2.00. Body slimmer than in *T.thoracica*. Posterior pronotal angles rectangular. Dorsal side rusty shiny, each elytron with indistinct apical and humeral pale rust spots, antennae rusty with antennomeres I, II and half of III dark yellow, legs dark yellow. Body length 2.00–2.23 mm	***T.sinaitica* (Schatzmayr, 1936)**
5(4)	Eyes more prominent, mean ratio of eye length / width 1.43. Body more robust than in *T.sinaitica*. Posterior pronotal angles obtuse. Coloration of dorsal side very variable, usually brown or dark brown, in dark specimens each elytron usually with apical and humeral rusty spots, in lighter specimens with distinct extensive light rusty apical and humeral spots, sometimes light rusty spots fused into longitudinal band (number of dark specimens in population increases towards north and to higher altitudes), antennae dark rusty or brown, antennomeres I, II and half of III dark yellow or pale rusty, legs dark yellow or pale rusty. Body length 2.00–2.45 mm	***T.thoracica* (Kolenati, 1845)**
6(3)	Each elytron with eight striae	**7**
7(8)	Elytral striae I–V and VIII well visible, with deep punctures, striae VI and VII formed only by punctures. Posterior pronotal angles rectangular. Eyes less prominent, mean ratio of eye length / width 1.67. Body slimmer than in *T.emerita*. Dorsal side rusty shiny; each elytron with indistinct apical and humeral pale rust spots, antennae rusty, antennomeres I, II and half of III pale rust, legs pale rusty. Body length 2.38–2.50 mm	***T.ferrugata* (Reitter, 1895)**
8(7)	Elytral striae I–IV with punctures, striae V–VIII formed only by punctures. Posterior pronotal angles obtuse. Eyes larger, more prominent, mean ratio of eye length / width 1.55. Body more robust than in *T.ferrugata*. Dorsal side dark yellow shiny, elytra without spots, antennae and legs dark yellow. Body length 1.83–2.08 mm	***T.emerita* (Péringuey, 1898)**

##### ﻿The varieties described by Johan Reinhold Sahlberg in Tachyina

Johan Reinhold Sahlberg (1903, 1913a, 1913b) described altogether six varieties in Tachyina. During a careful reading of Sahlberg’s papers we found that four of these varieties represent unavailable infrasubspecific names since a subspecies concept was used in the same works, and according to available information, these names were not validated prior to 1985 (International Commission on Zoological Nomenclature 1999, Article 45.6.4.1). The type specimens and one specimen of each species from the former Johan Reinhold Sahlberg collection are deposited in the Finnish Museum of Natural History (MZH) and the rest of his collection is deposited in the Zoological Museum of the University of Turku (Groll 2017). However, from the original material of the varieties described in Tachyina, we found only one specimen of Tachys (Tachyura) quadrisignatus var. caramanicus in MZH.

**Tachysscutellarisvar.flavicollis J.R. Sahlberg, 1903: 5**

An available name. A junior primary homonym of *Tachysflavicollis* Motschulsky, 1862. In catalogues it is usually listed as synonym of Tachys (Tachys) dimediatus dimediatus Motschulsky, 1849 (Kopecký 2003, 2017).

**Tachys (Tachyta) parvulus var. coarctatus J.R. Sahlberg, 1903: 5**

An available name. Sahlberg was evidently considering whether to describe this taxon as a separate species (“An species distincta?”) but ultimately chose to describe it as a new variety. In catalogues it is usually listed as synonym of Tachyura (Tachyura) parvula (Dejean, 1831) (Csiki 1928; Kopecký 2003, 2017).

**Tachys (Tachyura) sexstriatus var. brunneicollis J.R. Sahlberg, 1913b:19**

An unavailable infrasubspecific name since a subspecies concept was used in the same work and the name was not validated prior to 1985 (International Commission on Zoological Nomenclature 1999, Article 45.6.4.1). It was previously listed as synonym of *Tachyseuphratica* Reitter, 1885 (Kopecký 2017).

**Tachys (Tachyura) quadrisignatus var. caramanicus J.R. Sahlberg, 1913b:18**

An unavailable infrasubspecific name since a subspecies concept was used in the same work and the name was not validated prior to 1985 (International Commission on Zoological Nomenclature 1999, Article 45.6.4.1). It was listed by Kopecký (2017) as a subspecies of Tachyura (Tachyura) quadrisignata (Duftschmid, 1812).

**Tachysscutellarisvar.obscurus J.R. Sahlberg, 1913a:7**

**Tachysscutellarisvar.obscurus J.R. Sahlberg, 1913b:19**

Sahlberg (1913a, 1913b) proposed the name *obscurus* twice for the specimens from different localities. If they were available names, they would be homonyms. Because the subspecies concept was used in both works and the names were not validated prior to 1985 (International Commission on Zoological Nomenclature 1999, Article 45.6.4.1), they are infrasubspecific and unavailable. The first name was previously listed as a synonym of *Tachyscentromaculatus* Wollaston, 1864, and the second name as a homonym of T.scutellarisvar.obscurus J.R. Sahlberg, 1913a (Kopecký 2017).

## ﻿Discussion

In Tachyina, the shape of gonocoxites is a neglected character which has been described in only a few species of the genus *Tachyura* (Baehr 2016; Kopecký and Bezděk 2023). The preliminary results of the comparison of gonocoxites drawings of several dozen species in Tachyina show this character is useful to distinguish the species, and it is possible to trace common characteristics in genera and subgenera.

Checking the availability of the names of varieties described by J.R. Sahlberg for the present paper points to a wider problem that apparently has never been comprehensively resolved within Carabidae (at least not in Tachyina). Not all variety and form names meet the conditions of Articles 45.5 and 45.6 of the Code (International Commission on Zoological Nomenclature 1999). For example, when a fourth name follows a trinomen, that name is automatically infrasubspecific. Likewise, the name is deemed to be infrasubspecific if it was first published after 1960 and the author expressly used one of the terms variety or form. In many cases it is not easy to determine whether the name of the variety or form is of subspecific or infrasubspecific rank. A dichotomous key for the determination of rank was published by Lingafelter and Nearns (2013). A thorough evaluation of the validity of names can significantly clean the system from unavailable names, as has recently happened for example in the Palaearctic Chrysomelidae (Bezděk and Sekerka 2024).

## Supplementary Material

XML Treatment for Tachyura (s. str.) ferrugata

## ﻿Additional information

### Author ORCIDs

Tomáš Kopecký https://orcid.org/0009-0004-6314-1318

Jan Bezděk https://orcid.org/0000-0003-4358-7211

Jaakko Mattila https://orcid.org/0009-0002-2328-6227

### Data availability

All of the data that support the findings of this study are available in the main text.
